# The Development of Assays for Heparanase Enzymatic Activity: Towards a Gold Standard

**DOI:** 10.3390/molecules23112971

**Published:** 2018-11-14

**Authors:** Mohit Chhabra, Vito Ferro

**Affiliations:** 1 School of Chemistry and Molecular Biosciences, The University of Queensland, Brisbane, QLD 4072, Australia; m.chhabra@uq.edu.au; 2 Australian Infectious Diseases Research Centre, The University of Queensland, Brisbane, QLD 4072, Australia

**Keywords:** heparanase, enzyme assay, heparan sulfate, glycosaminoglycan, fondaparinux

## Abstract

The enzyme heparanase, an endo-β-glucuronidase, degrades heparan sulfate (HS) chains on the cell surface and in the extracellular matrix. Heparanase regulates numerous biological processes that drive tumour growth, metastasis and angiogenesis. In addition to its key role in cancer progression, it has also been implicated in an ever-growing number of other diseases, particularly those associated with inflammation. The importance of heparanase in biology has led to numerous efforts over the years to develop assays to monitor its activity and to screen for new inhibitors as potential drug candidates. Despite these efforts and the commercialization of a few kits, most heparanase assays are still complex, labour intensive, costly or have limited application. Herein we review the various methods for assaying heparanase enzymatic activity, focusing on recent developments towards new assays that hold the promise of accelerating research into this important enzyme.

## 1. Introduction

Mammalian heparanase is an endo-β-glucuronidase with both enzymatic and non-enzymatic functions. In addition to its important roles in physiology and development, heparanase mediates numerous pathological processes such as tumour growth, metastasis, angiogenesis and inflammation [1,2]. The primary enzymatic function of heparanase is to degrade heparan sulfate (HS), a highly sulfated polysaccharide found in the extracellular matrix (ECM) and on the mammalian cell surface [3]. The degradation of HS by heparanase regulates the bioavailability of HS-binding proteins, primes the tumour microenvironment and facilitates the spread of invasive tumour and inflammatory cells. Heparanase is now well established as a cancer drug target, and several inhibitors have progressed to clinical trials [4]. In recent years, heparanase has also been implicated in a range of other diseases [4], such as diabetes and its complications [5], kidney disease [6], atherosclerosis [7] and viral infections [8], to name a few, which continues to fuel research into this important protein. The potential for heparanase inhibitors as therapeutics for a range of diseases cannot be overstated.

Heparanase was recognised as a potential therapeutic target for cancer in the 1980s [9,10], but it was not until 1999 that it was first cloned and expressed [11,12]. The somewhat limited progress in heparanase research in the early years could be attributed in part to the lack of a robust, accurate and rapid assay for enzyme activity. Over the years, a plethora of assays have been developed to advance heparanase research and identify new inhibitors; however, no single method has emerged as the “gold standard”, despite the commercialization of a few assays. Indeed, the continued publication of new heparanase assays in the recent literature highlights the limitations of currently used methods and the need for improved assays. Herein, we first describe the substrate specificity and catalytic mechanism of heparanase, which are important considerations for assay design. We then review the various heparanase assays that have emerged over the years, focusing on recent developments and comment on their strengths and limitations. We have excluded assays for quantitation of heparanase expression levels or detection/visualization of heparanase localization in biological samples. Instead, we focus only on enzyme activity assays as the latter are of most interest for screening and identification of new inhibitors as potential therapeutics.

## 2. Heparanase Mechanism and Substrate Specificity

**Figure 1:**
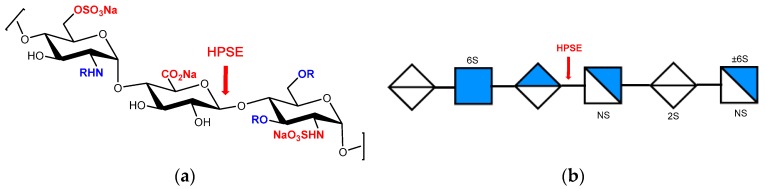
(**a**) Minimum HS substrate recognition sequence for heparanase. Essential charged groups in red; other charged groups which may improve affinity in blue (R = SO_3_Na); (**b**) reconstruction of the preferred HS cleavage sequences from a recent substrate specificity study [20].

Heparanase (HPSE), the sole HS-degrading endo-β-glucuronidase, is a member of the GH79 family of carbohydrate processing enzymes, which is characterised by a (β/α)8 domain containing the catalytic site [13]. The crystal structure of human heparanase has recently been solved, revealing details of its active site and HS-binding cleft [13]. Heparanase hydrolyses the internal glycosidic bonds of GlcA residues at a limited number of specific locations within HS to release smaller HS fragments. The hydrolysis occurs via a double displacement mechanism with net retention of anomeric configuration [14], and so heparanase is classed as a retaining glycosidase. Several substrate specificity studies [15,16,17,18,19,20] indicate that heparanase cleaves the linkage between a GlcA and an *N*-sulfoglucosamine residue carrying either a 3-*O*-sulfo or a 6-*O*-sulfo group. It was concluded that the minimum recognition sequence is a trisaccharide (Figure 1), consistent with recent findings from an X-ray crystallographic study [13]. These studies suggest that heparanase recognises certain sulfation patterns rather than specific monosaccharide sequences [18], and cleavage occurs at the non-reducing side of highly sulfated S domains [20]. Heparanase has also been shown to display plasticity in its substrate specificity depending on the structural features around the cleavage site [19,21].

## 3. Assaying Heparanase

Many and various assays for heparanase activity have been developed over the years. Most typically use HS, HS proteoglycan (HSPG) or heparin preparations as substrate and generally require prior labelling with radioisotopes (^35^S, ^14^C or ^3^H), fluorescent labels or biotin (or both) to allow detection. Many assays are microplate based in which HS is immobilised onto the surface of the plate either before or after treatment with heparanase. Heparanase activity is determined by quantifying either the smaller HS fragments liberated during catalysis or the residual unreacted substrate. Some of these plate-based assays have proven useful for high throughput screening, and a few have been commercialised. In recent years, there has been much interest in the development of heparanase assays using defined oligosaccharide substrates. For our discussion we have arbitrarily grouped these assays into those that use either heterogeneous heparin/HS based substrates or homogeneous HS oligosaccharide-based substrates.

### 3.1. Heparin/HS-Based Assays

An early assay used [^14^C]-labeled HS immobilised onto agarose gel beads [22], which resulted in release of labelled HS fragments upon treatment with heparanase. Similar assays were later developed using [^3^H]-labelled HS immobilised on sepharose beads and detected using 96-well plates [23], or with [^3^H]-labelled biotinylated HS immobilised on streptavidin-coated plates [24]. Another assay using [^3^H]-labelled HS passed the HS digest through a column of chicken histidine-rich glycoprotein (cHRG) immobilised on sepharose [25]. This assay takes advantage of the decreased affinity of the HS degradation products for cHRG.

Several heparanase assays were designed to fractionate between substrate and product HS based on their size difference. Early assays used size exclusion chromatography (SEC) [26,27] or SEC-HPLC [28] to separate between the two populations of radiolabelled HS. Later, ultrafiltration devices with specific pore size membranes were used to simplify and accelerate the process of fractionating digested HS [29,30,31]. Another assay takes advantage of the fact that the smaller HS products of heparanase catalysis are more soluble than undegraded HS in the presence of ionic detergents such as cetylpyridinium chloride, resulting in separation of substrate and product HS [28,32]. This assay is simple and inexpensive; however, inconsistency at the precipitation step reduces its robustness. Another size-dependent method for separating substrate HS from product that does not necessarily require radiolabelling is gel electrophoresis [33,34]. Size-dependent assays are typically longer and less suited to high throughput but have the benefits of being robust and do not rely upon expensive equipment.

In an effort to increase throughput, HS was incorporated within a tablet of polyacrylamide gel from which the smaller heparanase degraded fragments diffused leaving only the undegraded HS [34]. At the conclusion of this assay, Alcian Blue was used to quantify the remaining undegraded HS. The dye Tetrazolium Blue, which reacts with the reducing ends of carbohydrates, has also been used to assay heparanase activity, because with each catalytic event, a new reducing end is produced [35]. The simplicity and low cost are offset by the high background signal caused by the presence of reducing ends of the substrate HS which reduces the sensitivity.

**Figure 2:**
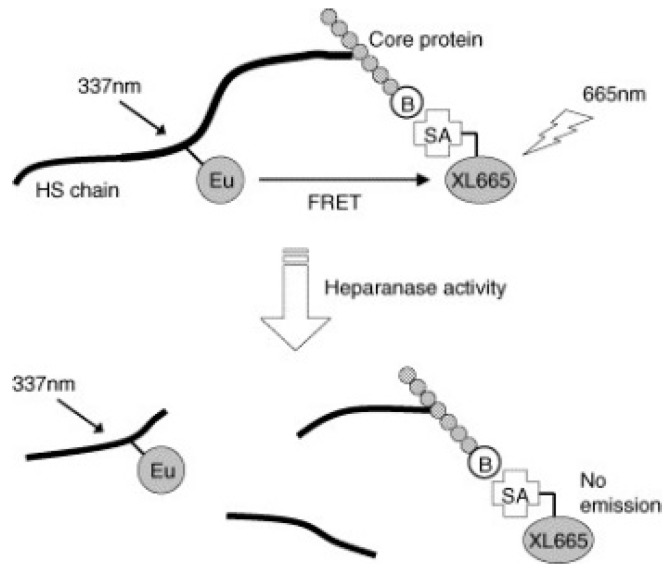
Heparanase assay using HTRF^®^. HSPG labelled with Eu-cryptate and biotin is detected by measuring time-resolved fluorescence after the addition of XL665-labeled streptavidin (XL665-SA). Degradation of substrate by heparanase decreases the HTRF signal. B, biotin; SA, streptavidin; FRET, fluorescence resonance energy transfer. Reproduced with permission [38].

Behzad and Brenchley biotinylated HS non-specifically with photobiotin and then formed reactive dialdehyde groups on the HS by treatment with sodium periodate. The substrate was then immobilised covalently onto a hydrazide-coated plate. Heparanase activity resulted in loss of the biotin label and was detected by a reduction in binding of streptavidin–horseradish peroxidase [36]. Huang et al. labelled HS at the reducing end with biotin followed by reaction with a fluorophore (FITC or a Eu-chelate). The dual-labelled HS substrate was then bound to streptavidin-coated plates, and the reaction products were monitored by the release of fluorescently labelled fragments in the solution [37]. Enomoto and coworkers developed a fluorescence resonance energy transfer (FRET)-based assay using a similarly dual-labelled HSPG substrate (Figure 2). The HS chain was labelled with a Eu-cryptate (fluorescence donor) via reductive amination onto dialdehyde groups generated by periodate treatment, and the core protein was labelled with biotin [38]. In this case, degradation of the substrate was detected by measuring the time-resolved fluorescence (homogeneous time-resolved fluorescence or HTRF^®^) following addition of streptavidin labelled with a cross-linked allophycocyanin pigment (XL665) as the fluorescence acceptor. This type of assay is available commercially [39]. Very recently, Desai and coworkers prepared a FRET substrate, heparin-DE, by incubating heparin simultaneously with the FRET pair of DABCYL and EDANS [40]. The substrate proved to be useful for screening inhibitors in a microplate format and has the advantage of rapid, one-step preparation. However, the product is heterogeneously labelled, making reproducibility an issue. In a similar, but more controlled, approach, Wang et al. [41] prepared a low molecular weight heparin (LMWH) substrate with the FRET donor and FRET acceptor exclusively at the non-reducing and reducing ends, respectively (see below).

Recently, biotinylated HS was immobilised onto a protamine-coated plate. Following treatment with heparanase, the immobilised biotinylated HS not digested by heparanase was detected with a Eu-streptavidin conjugate [42]. Wu and coworkers [43] biotinylated HS at the non-reducing end in an elaborate but controlled fashion by first enzymatically attaching an azido-labelled sugar, GlcNAz, with the glycosyltransferase EXT1/2 to HS chains attached to the proteoglycan rhSynd4. The biotin was introduced via a Cu(I)-catalysed “click” reaction, and the conjugate was then immobilised onto an anti-Synd4 antibody-coated plate. Detection of heparanase activity was via streptavidin-horseradish peroxidase, similar to Behzad and Brenchley. The above assays are similar in concept to some assays, including a commercially available kit [44], where the heparanase reaction is performed in solution, and the biotinylated HS is detected via avidin/streptavidin-peroxidase. In these assays, the digested HS is passed over a plate containing immobilised FGF-2 [44] or protamine [45] to which heparanase-degraded HS fragments do not bind. Detection in this type of assay has also been accomplished without biotinylation of HS by using a horseradish peroxidase-conjugated anti-FGF-2 antibody [46]. Another commercially available kit is presumably similar, although the identity of the HS-binding protein immobilised on the microplate is not revealed [47]. Huang et al. similarly used immobilised FGF-2 microplates with fluorescently labelled (FITC or Eu-chelate) but non-biotinylated HS with detection by fluorescence [37].

Heparanase is known to degrade the clinically used anticoagulants heparin and LMWH. Heparanase activity has thus also been determined indirectly by measuring the decrease in plasma anticogualant activity (APTT and anti-Xa activity) of heparin or LMWH [48]. Ahn and coworkers also demonstrated the use of an anti-Xa assay with LMWH as substrate but using bacterial heparinase I as a commercially available enzyme model for heparanase [49]. Some other assays for bacterial heparinases also have the potential to be utilised for heparanase but have yet to be fully exploited for this purpose. For example, a simple FRET-based substrate was recently reported by Wang et al. consisting of LMWH labelled with a fluorescent donor at the non-reducing end and an acceptor at the reducing end [41]. LMWH bearing a Δ4,5-unsaturated uronic acid at the non-reducing end was selectively labelled with 2-(2-mercaptoethylamino)benzamide via a Michael addition. The acceptor fluorophore, 2-aminoacridone, was then introduced at the reducing end via reductive amination. The substrate was successfully tested against bacterial heparinase II, but it should be applicable for assaying heparanase. Ban et al. [50] prepared HS-coated gold nanoparticles by first mixing HS in water with HAuCl_4_ followed by the addition of trisodium citrate. Cleavage of the HS chains by heparinase treatment resulted in the aggregation of the gold nanoparticles, causing a large redshift in the absorbance spectrum which was detectable to the naked eye. This assay should also be suitable for detecting heparanase activity. 

While some of the above HS-based assays offer improvements over past assays, overall the improvements have been incremental, and many of the assay principles can be considered to be simply variations on a theme. Some assays are undoubtedly useful for high throughput screening; however, they all have limitations. Other than the commercially available kits, most of the above methods are time consuming because of the need to label or immobilise the substrate. Radiolabelling has been largely superseded by labelling with biotin and/or fluorophores. However, the main limitations of using HS, other than cost, are common to most assays. HS is heterogeneous, making it difficult to standardise assays due to its high structural complexity and diversity. Moreover, HS contains multiple and variable heparanase cleavage sites, depending on the source of HS, and the assays are complicated by the fact that the heparin/HS fragments initially produced may either be substrates for further enzymatic cleavage, or inhibitors. The assays often only yield semi-quantitative results, usually as an IC_50_, making comparisons of the potency of various inhibitors from different sources difficult.

### 3.2. Homogeneous HS Oligosaccharide Substrates

**Scheme 1:**
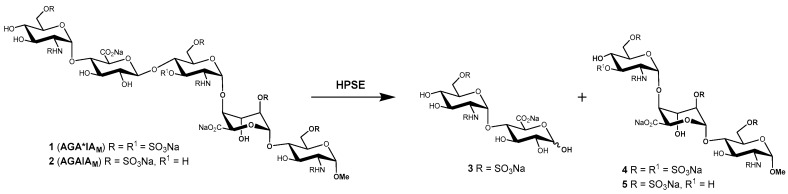
Cleavage of fondaparinux (**1**, AGA*IA_M_) and the related pentasaccharide (**2**, AGAIA_M_) by heparanase.

A homogeneous substrate of low molecular weight with a single point of cleavage clearly would greatly simplify assaying for heparanase activity. Homogeneous, low molecular weight heparin/HS oligosaccharides have indeed found great utility as tools and probes to understand the mechanism and substrate specificity of heparanase. Detection of heparanase activity has typically been carried out by SEC [15,19], HPLC [16], HPLC-MS [17] or NMR spectroscopy [14]. With the exception of fondaparinux (**1**, Scheme 1), most of these oligosaccharides have required either tedious separation and isolation following degradation of heparin/HS, sometimes with prior radiolabelling, or complex total or chemoenzymatic synthesis. Thus, they suffer from lack of (commercial) availability.

One homogeneous heparanase substrate that is commercially available is fondaparinux (**1**), the fully synthetic methyl glycoside of the antithrombin (AT)-activating pentasaccharide sequence of heparin [51], marketed as the anticoagulant drug Arixtra^®^. The cleavage of **1** with heparanase results in the formation of disaccharide **3** and trisaccharide **4** (Scheme 1), and the reaction can be readily followed by ^1^H-NMR spectroscopy [14]. A heparin-derived octasaccharide containing the AT binding sequence was shown to be cleaved by heparanase [15], and as discussed above, anti-Xa assays with heparin/LMWH heparin have been used to detect heparanase activity. Not surprisingly then, heparanase activity has also been determined by the anti-Xa assay using **1** as substrate [52,53], which should provide more consistent results compared with heparin/LMWH, which are heterogeneous and of variable composition. 

Bisio et al. developed an LC/MS method for detecting and quantifying the oligosaccharide cleavage products **3** and **4** following treatment of **1** (also known as AGA*IA_M_) with heparanase [17]. The LC/MS method is conceptually simple and has the advantage of directly quantifying the reaction products and not requiring the addition of exogenous protein (i.e., AT) which could potentially interfere with the assay. The method is suitable for screening potential heparanase inhibitors; however, the amount of protein consumed per assay is relatively high, and an unfavourable substrate to protein ratio make derivation of kinetic parameters difficult. In light of the above limitations, an improved assay was developed using **1** as the substrate based on the detection of the newly formed reducing disaccharide **3** [54] by reaction with the water-soluble tetrazolium salt 4-[3-(4-iodophenyl)-2-(4-nitrophenyl)-2*H*-5-tetrazolio]-1,3-benzene disulfonate (or WST-1). WST-1 reacts with the reducing end of disaccharide **3** to form a water-soluble formazan detected by UV absorbance at 584 nm, unlike other dyes which formed precipitates that interfered with the assay. The assay enabled the rapid kinetic characterization of inhibitors which were readily discriminated by their *K*_i_ values, with the data being directly comparable across studies. The simplicity and robustness of this method has seen it used successfully in drug discovery programs, e.g., leading to the discovery of the clinical candidate PG545 [55,56], and it has been miniaturized and used in a high-throughput screening campaign. Whilst useful for screening and characterizing inhibitors, a limitation of the assay is its unsuitability for assaying biological samples because of interference from other reducing compounds that also react with WST-1.

Recently, two new fondaparinux assays have been reported. The first uses “Polymer-H” [53,57], a fluorescent copolymer originally developed as a heparin sensor which strongly interacts with sulfated poly- and oligosaccharides, including **1**. When Polymer-H interacts with heparin or **1**, its fluorescence is quenched. However, oligosaccharides **3** and **4** have poor affinity for Polymer-H, so the cleavage of **1** by heparanase results in a measurable increase in fluorescence. This assay is more rapid than the WST-1 method and is not affected by the presence of reducing sugars in the sample; however, it is unclear whether it can be adapted to biological samples because of the presence of multiple endogenous glycosaminoglycans (GAGs) in the latter. The second assay is a variation of the WST-1 assay and uses the sodium salt of resazurin [42], a commercially available dye which also reacts with reducing sugars. Unlike WST-1, the product of the reaction is fluorescent and is detected at 590 nm following excitation at 560 nm. However, similarly to the WST-1 assay, it cannot be used for biological samples. 

**Figure 3:**
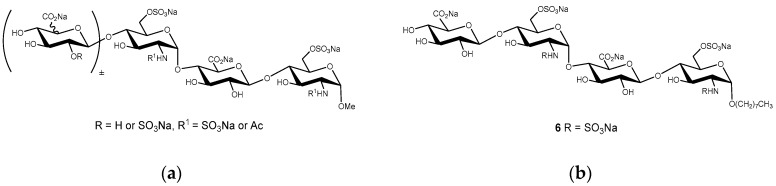
(**a**) Structures of putative heparanase substrates from a library synthesised by Yu and coworkers [59,61]; (**b**) putative heparanase tetrasaccharide substrate synthesised by Tamura and coworkers [60].

Whilst **1** is cleaved by heparanase, a better substrate is the related pentasaccharide **2** (AGAIA_M_) in which the 3-*O*-sulfo group of the central d-glucosamine residue is replaced by a hydroxy group. Compound **2** is hydrolysed approximately six times more rapidly [17] than **1**, in agreement with previous findings that 3-*O*-sulfation of the central d-glucosamine residue hinders cleavage by heparanase slightly [16]. However, **2** is only accessible via laborious total synthesis [58], whereas **1** is commercially available. Simpler HS tri- or tetrasaccharides have been synthesised as putative heparanase substrates (e.g., Figure 3) [59,60,61]. The degradation of these substrates by heparanase should be amenable to detection by similar means as described for **1**, e.g., via WST-1 or resazurin, but to date this has not been reported. Despite the shorter syntheses compared with **1**, the number of steps and overall yield further illustrate the challenges of total synthesis of heparin/HS oligosaccharides.

**Figure 4:**
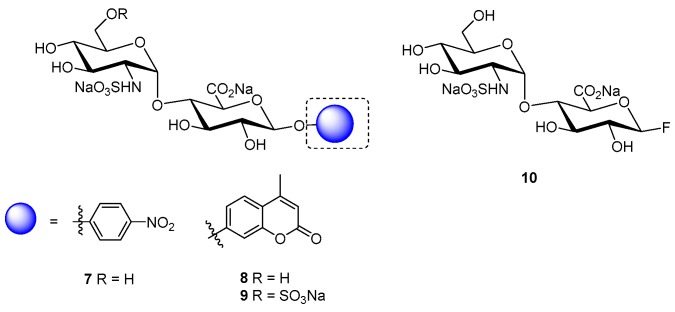
Structures of synthetic disaccharide substrates for heparanase [62,64].

The synthesis of simple, homogeneous heparanase substrates with chromogenic, fluorogenic or other non-sugar leaving groups has also attracted much interest. Fluorogenic and chromogenic substrates are commercially available and are used routinely for assaying numerous glycosidases. Such substrates are attractive because of their simplicity, reproducibility and ease of detection/sensitivity. In addition, they may be suitable for use in biological systems because of the lack of interference from endogenous molecules. Pearson et al. synthesised a series of disaccharides with various chromogenic or fluorogenic leaving groups as potential heparanase substrates [62]. Two of the *N*-sulfated compounds, **7** and **8** (Figure 4), with 4-nitrophenol and 4-methylumbelliferyl leaving groups, respectively, were shown to be cleaved by heparanase, although the specific activity was modest (17–48 nmol h^−1^ mg^−1^), especially for **7**. The corresponding *N*-acetylated compounds were inactive, as were those with charged aromatic aglycones. Compound **8** was the better substrate, offering greater sensitivity due to detection by fluorescence, and the 4-methylumbelliferyl is a better leaving group resulting in faster turnover. It was thus postulated that a compound such as **9**, with an additional 6-*O*-sulfo group on the glucosamine residue, as found in **1** or **2**, would likely have greater affinity for heparanase and would thus be a better substrate. Unfortunately, due to synthetic limitations, **9** was not prepared during this particular study. Recently, a molecular docking study of **9** using the X-ray crystal structure of recombinant human heparanase suggested that **9** should indeed be a useful substrate [63]. Disaccharide **9** has recently succumbed to synthesis (Ferro et al., unpublished results), and its performance as a heparanase substrate will be reported in due course.

Ohmae and coworkers [64] also reported a disaccharide substrate (**10**) for heparanase which is analogous to **8**. Compound **10** has a fluoride as the leaving group instead of a 4-methylumbelliferyl group, but also lacks 6-*O*-sulfation. Compound **10** was shown to be hydrolysed by heparanase via ^1^H-NMR spectroscopy. The reaction was relatively slow, with only 84% of substrate consumed after 6.6 h. This substrate would also likely benefit from an additional sulfo group at C-6 of the glucosamine residue.

## 4. Conclusions

The importance of heparanase in biology has led to the development of numerous assays over the years to detect its enzymatic activity and to screen for inhibitors. The assays developed to date have tried to address, mostly incrementally, the various limitations of their predecessors. Radiolabelling has largely been superseded by various colorimetric or fluorescence detection methods. Most assays continue to use heterogeneous substrates prepared by derivatization of HS or heparin/LMWH in various ways. These assays are limited by their heterogeneity and multiple enzyme cleavage sites but are often suitable for high throughput screening and are generally robust, allowing their use in biological samples. On the other hand, advances in the synthesis of simple synthetic oligosaccharide substrates with a single point of cleavage could ultimately lead to a “gold standard” assay for detailed kinetic analyses. Progress continues to be made with both HS and oligosaccharide-based assays, and it is anticipated that in the future more optimised assays will be commercially available. It remains to be seen whether a single, universal assay will emerge, or whether there will be different assays for different applications.

## References

[1] Sanderson R.D., Elkin M., Rapraeger A.C., Ilan N., Vlodavsky I. (2017). Heparanase regulation of cancer, autophagy and inflammation: New mechanisms and targets for therapy. FEBS J..

[2] Vlodavsky I., Singh P., Boyango I., Gutter-Kapon L., Elkin M., Sanderson R.D., Ilan N. (2016). Heparanase: From basic research to therapeutic applications in cancer and inflammation. Drug Resist. Updat..

[3] Li J.P., Kusche-Gullberg M. (2016). Heparan sulfate: Biosynthesis, structure, and function. Int. Rev. Cell Mol. Biol..

[4] Rivara S., Milazzo F.M., Giannini G. (2016). Heparanase: A rainbow pharmacological target associated to multiple pathologies including rare diseases. Future Med. Chem..

[5] Simeonovic C.J., Ziolkowski A.F., Wu Z., Choong F.J., Freeman C., Parish C.R. (2013). Heparanase and autoimmune diabetes. Front. Immunol..

[6] Rabelink T.J., van den Berg B.M., Garsen M., Wang G., Elkin M., van der Vlag J. (2017). Heparanase: Roles in cell survival, extracellular matrix remodelling and the development of kidney disease. Nat. Rev. Nephrol..

[7] Vlodavsky I., Blich M., Li J.P., Sanderson R.D., Ilan N. (2013). Involvement of heparanase in atherosclerosis and other vessel wall pathologies. Matrix Biol..

[8] Thakkar N., Yadavalli T., Jaishankar D., Shukla D. (2017). Emerging Roles of Heparanase in Viral Pathogenesis. Pathogens.

[9] Nakajima M., Irimura T., Di Ferrante D., Di Ferrante N., Nicolson G.L. (1983). Heparan sulfate degradation: Relation to tumor invasive and metastatic properties of mouse B16 melanoma sublines. Science.

[10] Vlodavsky I., Fuks Z., Bar-Ner M., Ariav Y., Schirrmacher V. (1983). Lymphoma cell-mediated degradation of sulfated proteoglycans in the subendothelial extracellular matrix: Relationship to tumor cell metastasis. Cancer Res..

[11] Hulett M.D., Freeman C., Hamdorf B.J., Baker R.T., Harris M.J., Parish C.R. (1999). Cloning of mammalian heparanase, an important enzyme in tumor invasion and metastasis. Nat. Med..

[12] Vlodavsky I., Friedmann Y., Elkin M., Aingorn H., Atzmon R., Ishai-Michaeli R., Bitan M., Pappo O., Peretz T., Michal I. (1999). Mammalian heparanase: Gene cloning, expression and function in tumor progression and metastasis. Nat. Med..

[13] Wu L., Viola C.M., Brzozowski A.M., Davies G.J. (2015). Structural characterization of human heparanase reveals insights into substrate recognition. Nat. Struct. Mol. Biol..

[14] Wilson J.C., Laloo A.E., Singh S., Ferro V. (2014). ^1^H-NMR spectroscopic studies establish that heparanase is a retaining glycosidase. Biochem. Biophys. Res. Commun..

[15] Pikas D.S., Li J.P., Vlodavsky I., Lindahl U. (1998). Substrate specificity of heparanases from human hepatoma and platelets. J. Biol. Chem..

[16] Okada Y., Yamada S., Toyoshima M., Dong J., Nakajima M., Sugahara K. (2002). Structural recognition by recombinant human heparanase that plays critical roles in tumor metastasis. Hierarchical sulfate groups with different effects and the essential target disulfated trisaccharide sequence. J. Biol. Chem..

[17] Bisio A., Mantegazza A., Urso E., Naggi A., Torri G., Viskov C., Casu B. (2007). High-performance liquid chromatographic/mass spectrometric studies on the susceptibility of heparin species to cleavage by heparanase. Semin. Thromb. Hemost..

[18] Peterson S.B., Liu J. (2010). Unraveling the specificity of heparanase utilizing synthetic substrates. J. Biol. Chem..

[19] Peterson S., Liu J. (2012). Deciphering mode of action of heparanase using structurally defined oligosaccharides. J. Biol. Chem..

[20] Mao Y., Huang Y., Buczek-Thomas J.A., Ethen C.M., Nugent M.A., Wu Z.L., Zaia J. (2014). A liquid chromatography-mass spectrometry-based approach to characterize the substrate specificity of mammalian heparanase. J. Biol. Chem..

[21] Peterson S.B., Liu J. (2013). Multi-faceted substrate specificity of heparanase. Matrix Biol..

[22] Nakajima M., Irimura T., Nicolson G.L. (1986). A solid-phase substrate of heparanase: Its application to assay of human melanoma for heparan sulfate degradative activity. Anal. Biochem..

[23] Xu Y.J., Miao H.Q., Pan W., Navarro E.C., Tonra J.R., Mitelman S., Camara M.M., Deevi D.S., Kiselyov A.S., Kussie P. (2006). *N*-(4-{[4-(1H-Benzoimidazol-2-yl)-arylamino]-methyl}-phenyl)-benzamide derivatives as small molecule heparanase inhibitors. Bioorg. Med. Chem. Lett..

[24] Nardella C., Steinkuhler C. (2004). Radiolabeled heparan sulfate immobilized on microplate as substrate for the detection of heparanase activity. Anal. Biochem..

[25] Freeman C., Parish C.R. (1997). A rapid quantitative assay for the detection of mammalian heparanase activity. Biochem. J..

[26] Bar-Ner M., Eldor A., Wasserman L., Matzner Y., Cohen I.R., Fuks Z., Vlodavsky I. (1987). Inhibition of heparanase-mediated degradation of extracellular matrix heparan sulfate by non-anticoagulant heparin species. Blood.

[27] Nakajima M., DeChavigny A., Johnson C.E., Hamada J., Stein C.A., Nicolson G.L. (1991). Suramin. A potent inhibitor of melanoma heparanase and invasion. J. Biol. Chem..

[28] Lapierre F., Holme K., Lam L., Tressler R.J., Storm N., Wee J., Stack R.J., Castellot J., Tyrrell D.J. (1996). Chemical modifications of heparin that diminish its anticoagulant but preserve its heparanase-inhibitory, angiostatic, anti-tumor and anti- metastatic properties. Glycobiology.

[29] Tsuchida S., Podyma-Inoue K.A., Yanagishita M. (2004). Ultrafiltration-based assay for heparanase activity. Anal. Biochem..

[30] Karoli T., Liu L., Fairweather J.K., Hammond E., Li C.P., Cochran S., Bergefall K., Trybala E., Addison R.S., Ferro V. (2005). Synthesis, Biological Activity, and Preliminary Pharmacokinetic Evaluation of Analogues of a Phosphosulfomannan Angiogenesis Inhibitor (PI-88). J. Med. Chem..

[31] Fairweather J.K., Hammond E., Johnstone K.D., Ferro V. (2008). Synthesis and heparanase inhibitory activity of sulfated mannooligosaccharides related to the antiangiogenic agent PI-88. Bioorg. Med. Chem..

[32] Bame K.J., Danda J., Hassall A., Tumova S. (1997). Aβ(1-40) prevents heparanase-catalyzed degradation of heparan sulfate glycosaminoglycans and proteoglycans in vitro. A role for heparan sulfate proteoglycan turnover in Alzheimer’s disease. J. Biol. Chem..

[33] De Vouge M.W., Yamazaki A., Bennett S.A., Chen J.H., Shwed P.S., Couture C., Birnboim H.C. (1994). Immunoselection of GRP94/endoplasmin from a KNRK cell-specific lambda gt11 library using antibodies directed against a putative heparanase amino-terminal peptide. Int. J. Cancer.

[34] Ishida K., Teruya T., Simizu S., Osada H. (2004). Exploitation of heparanase inhibitors from microbial metabolites using an efficient visual screening system. J. Antibiot..

[35] Ben-Artzi H., Ayal-Hershkovitz M., Vlodavsky I., Pecker I., Peleg Y., Miron D. (2001). Method of Screening for Potential Anti-Metastatic and Anti-Inflammatory Agents Using Mammalian Heparanase as a Probe. US Patent.

[36] Behzad F., Brenchley P.E. (2003). A multiwell format assay for heparanase. Anal. Biochem..

[37] Huang K.S., Holmgren J., Reik L., Lucas-McGady D., Roberts J., Liu C.M., Levin W. (2004). High-throughput methods for measuring heparanase activity and screening potential antimetastatic and anti-inflammatory agents. Anal. Biochem..

[38] Enomoto K., Okamoto H., Numata Y., Takemoto H. (2006). A simple and rapid assay for heparanase activity using homogeneous time-resolved fluorescence. J. Pharm. Biomed. Anal..

[39] Cisbio Heparanase Assay Toolbox. https://www.cisbio.com/drug-discovery/heparanase-assay-toolbox.

[40] Sistla J.C., Morla S., Alabbas A.-H.B., Kalathur R.C., Sharon C., Patel B.B., Desai U.R. (2019). Polymeric fluorescent heparin as one-step FRET substrate of human heparanase. Carbohydr. Polym..

[41] Wang Z.Q., Shi C., Wu X.R., Chen Y.J. (2014). Efficient access to the non-reducing end of low molecular weight heparin for fluorescent labeling. Chem. Commun..

[42] Melo C.M., Tersariol I.L., Nader H.B., Pinhal M.A., Lima M.A. (2015). Development of new methods for determining the heparanase enzymatic activity. Carbohydr. Res..

[43] Wu Z.L., Huang X., Ethen C.M., Tatge T., Pasek M., Zaia J. (2017). Non-reducing end labeling of heparan sulfate via click chemistry and a high throughput ELISA assay for heparanase. Glycobiology.

[44] TaKaRa Bio Heparan Degrading Enzyme Assay Kit. https://www.takarabio.com.

[45] Haubeck H.D. (2008). Method for Determining Endoglycosidase (Heparanase) Enzyme Activity. PCT Int. Appl..

[46] Courtney S.M., Hay P.A., Buck R.T., Colville C.S., Porter D.W., Scopes D.I.C., Pollard F.C., Page M.J., Bennett J.M., Hircock M.L. (2004). 2,3-Dihydro-1,3-dioxo-1H-isoindole-5-carboxylic acid derivatives: A novel class of small molecule heparanase inhibitors. Bioorg. Med. Chem. Lett..

[47] Galantos Galantos Heparanase Assay. https://www.galantos.eu/heparanase/pdf/galantos_broschuere_hepa2010.pdf.

[48] Nasser N.J., Sarig G., Brenner B., Nevo E., Goldshmidt O., Zcharia E., Li J.P., Vlodavsky I. (2006). Heparanase neutralizes the anticoagulation properties of heparin and low-molecular-weight heparin. J. Thromb. Haemost..

[49] Ahn S.C., Kim B.Y., Oh W.K., Park Y.M., Kim H.M., Ahn J.S. (2006). Colorimetric heparinase assay for alternative anti-metastatic activity. Life Sci..

[50] Ban Z., Bosques C.J., Sasisekharan R. (2008). A simple assay to probe disease-associated enzyme activity using glycosaminoglycan-assisted synthesized gold nanoparticles. Org. Biomol. Chem..

[51] Petitou M., van Boeckel C.A. (2004). A synthetic antithrombin III binding pentasaccharide is now a drug! What comes next?. Angew. Chem. Int. Ed. Engl..

[52] Driguez P.A., Petitou M. (2006). Azasugar Derivatives, Heparanase Inhibitors, Method for Preparing Same, Compositions Containing Same, Use Thereof. PCT Int. Appl..

[53] Schiemann S., Luhn S., Alban S. (2012). Development of both colorimetric and fluorescence heparinase activity assays using fondaparinux as substrate. Anal. Biochem..

[54] Hammond E., Li C.P., Ferro V. (2010). Development of a colorimetric assay for heparanase activity suitable for kinetic analysis and inhibitor screening. Anal. Biochem..

[55] Dredge K., Hammond E., Davis K., Li C.P., Liu L., Johnstone K., Handley P., Wimmer N., Gonda T.J., Gautam A. (2010). The PG500 series: Novel heparan sulfate mimetics as potent angiogenesis and heparanase inhibitors for cancer therapy. Invest. New Drugs.

[56] Ferro V., Liu L., Johnstone K.D., Wimmer N., Karoli T., Handley P., Rowley J., Dredge K., Li C.P., Hammond E. (2012). Discovery of PG545: A Highly Potent and Simultaneous Inhibitor of Angiogenesis, Tumor Growth, and Metastasis. J. Med. Chem..

[57] Schoenfeld A.K., Vierfuss S., Luhn S., Alban S. (2014). Testing of potential glycan-based heparanase inhibitors in a fluorescence activity assay using either bacterial heparinase II or human heparanase. J. Pharm. Biomed. Anal..

[58] Petitou M., Duchaussoy P., Lederman I., Choay J., Sinay P. (1988). Binding of heparin to antithrombin III: A chemical proof of the critical role played by a 3-sulfated 2-amino-2-deoxy-d-glucose residue. Carbohydr. Res..

[59] Chen J., Zhou Y., Chen C., Xu W., Yu B. (2008). Synthesis of a tetrasaccharide substrate of heparanase. Carbohydr. Res..

[60] Takeda N., Ikeda-Matsumi R., Ebara-Nagahara K., Otaki-Nanjo M., Taniguchi-Morita K., Nanjo M., Tamura J. (2012). Synthesis of heparan sulfate tetrasaccharide as a substrate for human heparanase. Carbohydr. Res..

[61] Xu P., Xu W., Dai Y., Yang Y., Yu B. (2014). Efficient synthesis of a library of heparin tri- and tetrasaccharides relevant to the substrate of heparanase. Org. Chem. Front..

[62] Pearson A.G., Kiefel M.J., Ferro V., von Itzstein M. (2011). Synthesis of simple heparanase substrates. Org. Biomol. Chem..

[63] Pennacchio A., Capo A., Caira S., Tramice A., Varriale A., Staiano M., D’Auria S. (2018). Cloning and bacterial expression systems for recombinant human heparanase production: Substrate specificity investigation by docking of a putative heparanase substrate. Biotechnol. Appl. Biochem..

[64] Ohmae M., Fujita Y., Takada J., Kimura S. (2013). Synthesis of a Heparan Sulfate Disaccharide Fluoride for Detection of Heparanase Activity. Chem. Lett..

